# LINKS OF SLEEP WITH CHANGES IN CARDIORESPIRATORY FITNESS AND ENERGETIC EFFICIENCY IN MIDDLE-AGED AND OLDER ADULTS

**DOI:** 10.1093/geroni/igae098.1199

**Published:** 2024-12-31

**Authors:** Yiwei Yue, Daniel Callow, Idiatou Diallo, Jill Rabinowitz, Sarah Wanigatunga, Eleanor Simonsick, Jennifer Schrack, Adam Spira

**Affiliations:** Johns Hopkins University, Baltimore, Maryland, United States; Johns Hopkins University School of Medicine, Baltimore, Maryland, United States; Johns Hopkins University, Baltimore, Maryland, United States; Rutgers, The State University of New Jersey, New Brunswick, New Jersey, United States; Johns Hopkins University, Baltimore, Maryland, United States; National Institute on Aging, Baltimore City 0400 Baltimore City, Maryland, United States; Johns Hopkins Bloomberg School of Public Health, Baltimore, Maryland, United States; Johns Hopkins University, Baltimore, Maryland, United States

## Abstract

Sleep disturbances are linked to cardiovascular disease in middle-aged and older adults, but little is known about the associations of poor sleep with cardiovascular fitness and energetic measures in this population. We investigated cross-sectional and longitudinal associations of actigraphic sleep parameters with cardiorespiratory fitness, walking energetics, and resting metabolic rate among 263 participants (mean age=72.7±10.1 years, 53.6% women) in the Baltimore Longitudinal Study of Aging. Additionally, we assessed modifying roles of sex, race, and age in these associations. Primary predictors included actigraphic total sleep time (TST), sleep efficiency (SE; % of time in bed spent asleep), sleep onset latency (SOL), wake after sleep onset (WASO), and average wake bout length (WBL). Outcomes included maximal oxygen consumption (VO2max), energetic cost of walking (ECW), and resting metabolic rate (RMR). Adjusting for demographics, comorbidities, self-reported physical activity, time, and interactions of time with covariates, longer WBL was cross-sectionally associated with lower VO2max (B=-1.24, p<0.01) and higher RMR (B=62.65, p<0.01); lower SE with lower VO2max (B=1.18, p<0.01); and shorter TST with higher RMR (B=-16.03, p<0.05). Women had stronger associations between longer TST and higher VO2max and greater WBL with slower decline in VO2max than men. Middle-aged adults showed stronger connections of higher SE and lower WASO with lower RMR than older adults (interaction p-values<0.05). In summary, shorter TST, longer WBL, and lower SE are linked with poorer cardiorespiratory fitness and energetic efficiency among middle-aged and older adults. Studies are needed to determine whether enhancing sleep can improve fitness and energetic efficiency, and vice-versa.

